# The Effect of Night Shifts on 24-h Rhythms in the Urinary Metabolome of Police Officers on a Rotating Work Schedule

**DOI:** 10.1177/07487304221132088

**Published:** 2022-11-08

**Authors:** Laura Kervezee, Anna Koshy, Nicolas Cermakian, Diane B. Boivin

**Affiliations:** * Centre for Study and Treatment of Circadian Rhythms, Douglas Mental Health University Institute, Department of Psychiatry, McGill University, Montreal, QC, Canada; † Laboratory of Molecular Chronobiology, Douglas Mental Health University Institute, Department of Psychiatry, McGill University, Montreal, QC, Canada; ‡ Laboratory for Neurophysiology, Department of Cellular and Chemical Biology, Leiden University Medical Center, Leiden, the Netherlands

**Keywords:** circadian rhythm, metabolomics, night shift work, occupational health, urine

## Abstract

Shift workers face an increased risk of metabolic health problems, but the direct metabolic response to working nights is not fully understood. The aim of this study was to investigate the effect of night shifts on the 24-h urinary metabolome of shift workers. Eleven police officers working rotating shifts completed two 24-h laboratory visits that took place before and after they worked 7 consecutive nights. Sleep and meals were scheduled on a day schedule in the first visit and then on a night schedule (i.e., sleep and meals shifted by approximately 12 h) in the second visit. Targeted metabolomic analysis was performed on urine samples collected throughout these laboratory visits. Differential rhythmicity analysis was used to compare 24-h rhythms in urinary metabolites in both conditions. Our results show that on the day schedule, 24-h rhythms are present in the urinary levels of the majority of metabolites, but that this is significantly reduced on the night schedule, partly due to loss of organic acid rhythmicity. Furthermore, misalignment of 24-h metabolite rhythms with the shifted behavioral cycles in the night schedule was observed in more than half of the metabolites that were rhythmic in both conditions (all acylcarnitines). These results show that working nights alters the daily rhythms of the urinary metabolome in rotating shift workers, with the most notable impact observed for acylcarnitines and organic acids, 2 metabolite classes involved in mitochondrial function. Further research is warranted to study how these changes relate to the increased metabolic risks associated with shift work.

Night shift work is associated with an increased risk of various adverse metabolic health effects such as cardiovascular diseases, metabolic syndrome, type 2 diabetes, and obesity (Gan et al., 2015; Sun et al., 2018; Torquati et al., 2018; Yang et al., 2021). With around 20%-30% of the workforce in industrialized countries involved in atypical working schedules (Kecklund and Axelsson, 2016), the factors that underlie these health issues warrant attention from a public health point of view.

The misalignment between timing cues from the environment and internal physiological rhythms that are coordinated by the endogenous circadian system is thought to contribute to the adverse health effects associated with shift work (Finger and Kramer, 2021; Boivin et al., 2022). Controlled laboratory studies in healthy human subjects have shown that circadian misalignment for as little as a few days leads to marked physiological changes, including impaired glucose metabolism, altered endocrine and immune responses, increased blood pressure, and elevated levels of inflammatory markers (Scheer et al., 2009; Morris et al., 2015; Cuesta et al., 2016; Morris et al., 2016a; Bescos et al., 2018; Wefers et al., 2018; Ruiz et al., 2020). Reduced glucose tolerance in response to a 3-day intervention to induce circadian misalignment has also been observed in real shift workers, providing a potential link between circadian misalignment and diabetes risk in shift workers (Morris et al., 2016b).

Metabolomic studies can provide a detailed biochemical snapshot of the cellular processes in the body and have the potential to shed light on the metabolic response to lifestyle or occupational factors, such as night shifts. Various controlled laboratory studies have characterized daily rhythms in the human metabolome in multiple tissues and biofluids, including plasma, breath, saliva, skeletal muscle, and urine, revealing that the metabolome displays significant changes over the course of the 24-h period (Dallmann et al., 2012; Davies et al., 2014; Martinez-Lozano Sinues et al., 2014; Giskeodegard et al., 2015; Loizides-Mangold et al., 2017; Held et al., 2020). These 24-h rhythms could be driven by the circadian timing system as well as by exogenous factors such as sleep and feeding (Hancox et al., 2021; Nowak et al., 2021). The response to night shifts has also been studied in controlled laboratory studies, revealing that 24-h rhythms in circulating metabolites adapt to the shifted behavioral cycles of feeding and fasting within a few days and therefore become uncoupled from central and peripheral circadian clocks that take longer to adapt (Skene et al., 2018; Kervezee et al., 2019). Studies on the metabolomic response to night shifts in real shift workers are scarce. Rotter et al. (2018) performed targeted metabolomic analysis on post-waking urine samples (i.e., 1 time point) from female nurses working day and night shifts and found increased levels of acylcarnitines during night shifts, possibly reflecting changes in fatty acid oxidation. However, it is unknown whether, and to what extent, night shift work affects 24-h profiles of metabolites. Therefore, the goal of this study was to characterize 24-h urinary metabolite profiles before and after 7 days of night shifts in rotating shift workers to shed light on the acute metabolomic changes associated with night shift work.

## Materials and Methods

### Participants and Study Design

The current study is based on the analysis of samples collected in an observational study on the adaptation of central and peripheral clocks in shift workers that was previously published, in addition to details on recruitment and screening of participants, as well as the experimental protocol (Koshy et al., 2019). Briefly, participants (both men and women) were recruited from a group of police officers who participated in a larger field study on the effects of rotating shift work, the results of which are described elsewhere (Kervezee et al., 2021). Men and women within the age range of 20-67 years were considered for inclusion. Participants were required to be mentally and physically fit. Exclusion criteria included being diagnosed for a sleep disorder unrelated to their work schedule, as well as existence of seasonal affective disorder (screened with the Seasonal Pattern Assessment Questionnaire) and sleep apnea (screened with the Berlin Questionnaire). The study was approved by the Douglas Institute Ethics Board (protocol number: IUSMD-1434) and was conducted according to ethical standards of the Declaration of Helsinki. All subjects provided written informed consent prior to the study. Participants were included over the period of 1 year, from February 2016 to January 2017. The laboratory visits took place in the research facility of the Centre for Study and Treatment of Circadian Rhythms at the Douglas Mental Health University Institute in Montreal, Canada. Because this was an exploratory study based on the analysis of samples collected for a different primary objective, no formal power analysis was performed.

**Figure 1.**
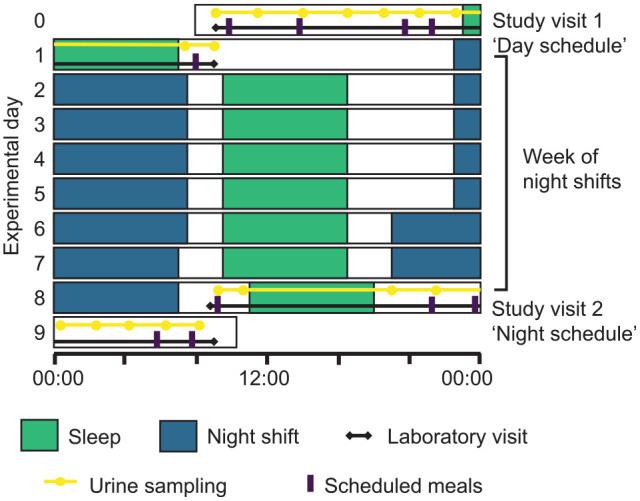
Study protocol. Participants entered the laboratory on day 0 for study visit 1, during which they lived on a day schedule with the sleep period scheduled from 2300 to 0700 h. Urine samples were collected at predetermined time points and additional samples were collected in case of additional voids. From day 1 to day 8, participants completed a week of night shifts as part of their usual work roster. The timing of daytime sleep episodes following night shifts is approximate. After the final night shift on day 8, participants returned to the laboratory to complete study visit 2, during which they lived on a night schedule with the sleep period scheduled from 1100 to 1900 h.

The study protocol consisted of two 24-h laboratory visits that took place before and after participants worked 7 consecutive night shifts as part of their regular work schedule (Figure 1). Prior to the first laboratory visit, participants lived on a day schedule for at least 1 week, during which they worked morning and evening shifts and had days off. Following this study visit, they worked 7 consecutive nights, consisting of five 9-h shifts followed by two 12-h shifts (see Koshy et al., 2019 for details). Immediately following the last night shift, they entered the laboratory for their second study visit, during which participants lived on a night schedule.

Both laboratory visits started around 0900 h and ended around 1000 h the next morning. During these visits, the police officers were admitted to a time isolation room, where activity levels were kept to a minimum. An 8-h sleep period was scheduled from 2300 to 0700 h during the first study visit and from 1100 to 1900 h during the second study visit. Light levels were below 275 lux during the wake period in the laboratory and ~0 lux during scheduled sleep periods in the laboratory. Meal times were shifted by approximately 12 h during the second visit (day schedule visit: breakfast at 0945 h, lunch at 1345 h, dinner at 1945 h, a snack at 2110 h, and breakfast the next day at 0810 h; night schedule visit: snack at 0902 h, breakfast at 2110 h, lunch at 2345 h, dinner next day at 0545 h, and a snack at 0745 h). Water was available ad libitum.

### Urine Sampling and Processing

During the laboratory visits, all urine provided by the participants was collected. Participants provided urine samples before and after sleep periods and every 2-3 h during wake periods. When needed, participants could provide additional urine samples outside these scheduled time points. Exact times and volume of all voids were recorded. At each time of collection, 2 aliquots of 3.5 mL each were taken and stored at −20 °C until further analysis, and the remainder of the sample was discarded. Long-term storage of urine samples at −20 °C has been shown to have a minimal (if any) effect on the metabolomic profile compared with storage at −80 °C (Laparre et al., 2017; Stevens et al., 2019). Samples (single aliquots) were thawed only once immediately prior to metabolomic profiling. The exact voiding times were used to calculate the midpoint of the urine collection periods. Midpoints were used as the time variable in subsequent analyses.

Urinary flow rate (in L/h) was calculated for each void by dividing the volume of the collected urine by the time between 2 voids. As the time of voiding prior to entry into the laboratory was not documented, urinary flow rate could not be calculated for the first urine sample. As the urinary flow rate was used to normalize metabolite concentrations (see below), the first sample was not used for metabolomic profiling.

### Metabolomic Profiling

Targeted, quantitative metabolomic analysis using a combination of direct injection mass spectrometry with a reverse-phase liquid chromatography-mass spectrometry (LC-MS)/MS was performed by the Metabolomics Innovation Centre (TMIC) at the University of Alberta in Edmonton (Canada) (Bouatra et al., 2013). Sample preparation and mass spectrometric analysis were performed using an in-house, validated assay as described previously (Zheng et al., 2020). Metabolite quantification was done using Analyst 1.6.2.

### Data Processing

All subsequent data processing and statistical analyses were performed in R v4.0.3 (R Core Team, 2020). Metabolites were excluded from further analysis if their concentrations were below the limit of quantification in more than 10% of the samples. For the remaining metabolites, concentrations that were missing because they were below the limit of detection were set to either half the minimum concentration of that metabolite in our dataset or half the limit of quantification (whichever value was lower) (Xia et al., 2009). Visual inspection of principal component analysis by sample, in combination with experimental notes, was used to identify and exclude sample outliers. To account for variations in urine volume, metabolite concentrations (in μmol/L) were normalized by urinary flow rate (in L/h; see above) prior to statistical analysis, yielding metabolite excretion rates in μmol/h (also referred to as metabolite levels).

### Statistical Analysis

Metabolite levels were log-transformed prior to analyses to reduce heteroscedasticity and skewness (van den Berg et al., 2006). To characterize 24-h rhythms in metabolite levels and identify increases or decreases in the average 24-h levels during the study visits, differential rhythmicity analysis using a model selection approach was performed as described previously (Kervezee et al., 2018). Because this method can handle unevenly spaced repeated measures, no imputation was performed for missing time points (see below). This analysis was performed using linear mixed-effects models with participant included as a random effect using R package lme4, version 1.1-25 (Bates et al., 2015). Bayesian Information Criterion (BIC) weights were used to probabilistically assign metabolites to 1 of 10 possible model categories. For each metabolite, the model with the highest BIC weight was selected if it exceeded the threshold of 0.4 (Kervezee et al., 2018). Using this approach, metabolites were classified as rhythmic with shared (models i and vi) or different (models ii and vii) cosinor coefficients (i.e., amplitude and peak timing) during the day and night schedules; as rhythmic only during the day schedule (models iii and viii), as rhythmic only during the night schedule (models iv and ix), or as not rhythmic in either condition (models v and x); and with either a shared mesor (i.e., similar average 24-h levels; models i, ii, iii, iv, and v) or a different mesor (higher or lower average levels; models vi, vii, viii, ix, and x) before and after night shifts. To assess significance, the fit of the selected model was compared with the null model (model v) using a log-likelihood ratio test. *P* values were corrected for multiple testing using the Benjamini-Hochberg method (false discovery rate [FDR] < 0.05). Linearized cosinor analysis was performed on metabolites classified as rhythmic by the differential rhythmicity analysis, and the cosinor coefficients were used to calculate the amplitude and peak timing of these metabolites (Cornelissen, 2014). To determine whether metabolite classes (i.e., acylcarnitines, amino acids, biogenic amines, organic acids, and others) were overrepresented relative to other metabolite classes among metabolites in the different model categories, Fisher exact tests were performed. Overrepresentation was considered significant if *p* < 0.05. Statistical significance of the difference in the amplitude of rhythmic metabolites between the day schedule and the night schedule was computed using a linear mixed-effects model (“metabolite” included as random effect) using the R package lme4 (Bates et al., 2015). Prior to visualization of data in heatmaps, log-transformed metabolite values were *z*-scored by centering (i.e., subtracting the mean metabolite levels) and scaling (i.e., dividing by the standard deviation).

## Results

Demographic details of the study population and the study protocol were published previously (Koshy et al., 2019). Briefly, 11 police officers were included in the study (7 males, 4 females), aged 28 ± 3 (mean ± SD; range: 24-34) years and having a body mass index of 24.1 ± 2.3 kg/m^2^. In total, 190 urine samples were collected during the study visits and used for targeted metabolomic analysis (Supplemental Figure S1A). Three urine samples were excluded from further analysis: 2 of these samples had an extremely low volume (<10 mL), and the third sample also had a relatively low volume (29 mL) and the experimental notes revealed a protocol deviation. These samples were also identified as outliers upon visual inspection of principal component analysis based on the normalized metabolomic data (Supplemental Figure S1B). Therefore, 187 urine samples were included in subsequent analyses: 94 samples were collected during the day-oriented study visit and 93 samples were collected during the night-oriented study visit (range: 7-11 samples per visit per participant).

### Characterization of 24-h Rhythms in Urinary Metabolites

A total of 138 metabolites were assayed, of which 59 metabolites were subsequently excluded because their concentrations were below the limit of quantification in more than 10% of the samples. The excluded metabolites mainly included long-chain acylcarnitines and lipids, which are known to have very low concentrations in urine (Bouatra et al., 2013). The 79 metabolites included in the analysis comprised 20 acylcarnitines, 22 amino acids, 17 biogenic amines, and 17 organic acids, as well as glucose, choline, and trimethylamine N-oxide (TMAO).

**Figure 2.**
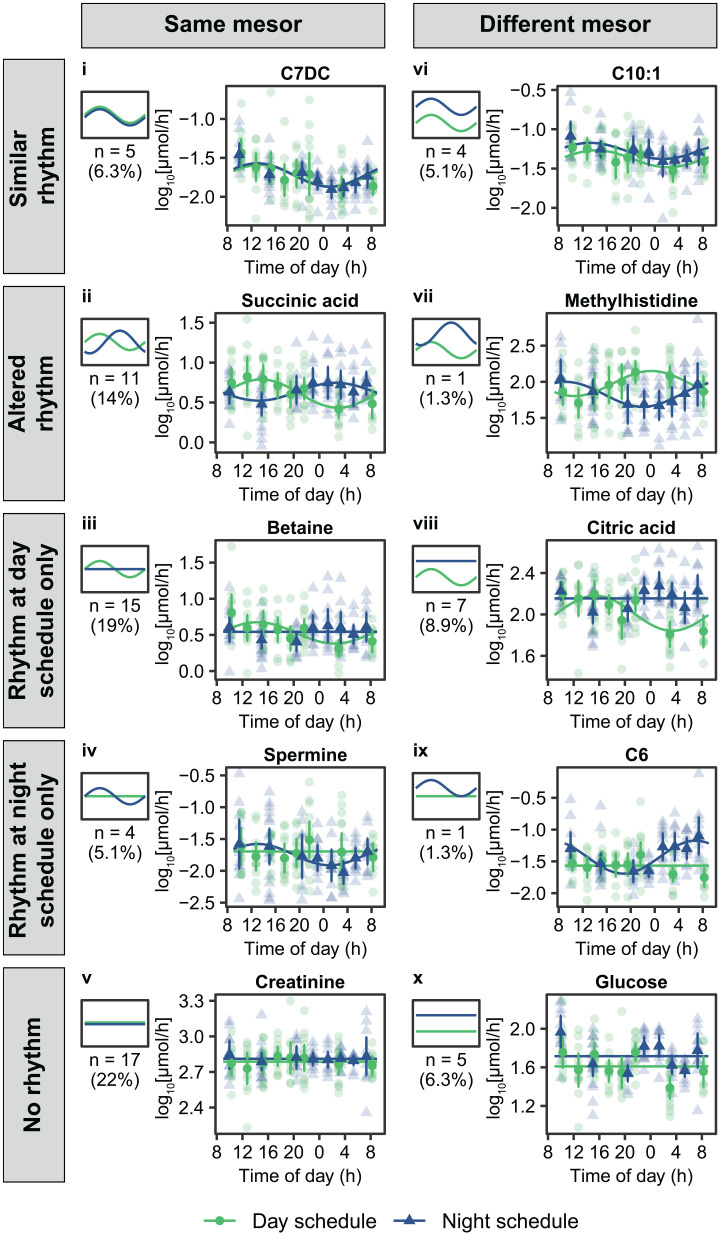
Representative examples of metabolites assigned to 1 of 10 categories using differential rhythmicity analysis. Different panels (numbered i-x) represent the different categories. Small plots on the left of each panel provide a schematic representation of each model category and plots on the right show the 24-h profiles during the day schedule (green symbols and lines) and the night schedule (blue symbols and lines) for representative metabolites within each category. Semi-transparent data points represent individual metabolite levels, and fully opaque data points and error bars represent the mean ± 95% confidence intervals. Solid lines represent model predictions for each metabolite.

Differential rhythmicity analysis was used to classify metabolites in 10 categories based on their changes in overall excretion rates (i.e., change in mesor) and in 24-h rhythms (i.e., peak timing or amplitude) during the night schedule compared with the day schedule. Representative examples of metabolites for each model category are shown in Figure 2. Seventy metabolites (89%) could be assigned to 1 of the 10 categories, and the remaining 9 metabolites were classified as ambiguous (BIC weight <0.4) (Suppl. Data S1). All selected models provided a significantly better fit to the data compared with a null model (model v in Figure 2) with similar mesors for both conditions and no 24-h cosinor function included (all FDR-adjusted *p* values <0.013).

**Figure 3.**
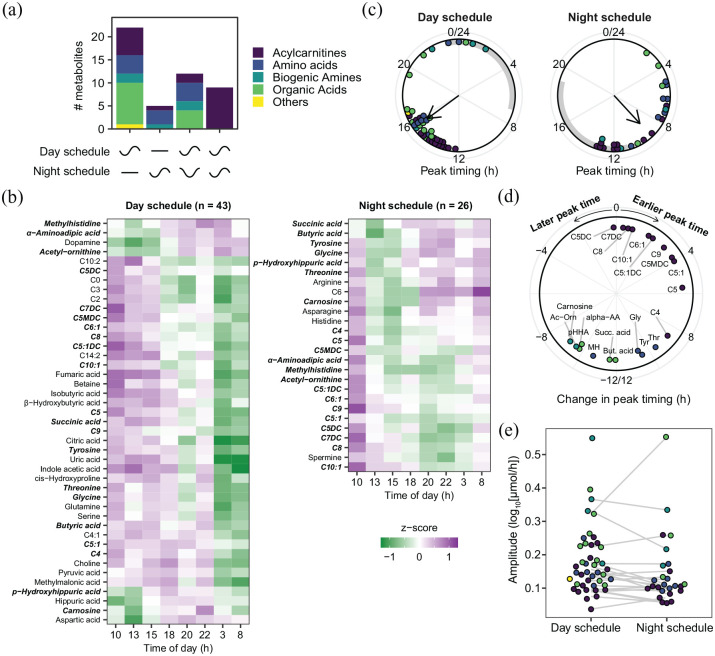
Twenty-four-hour rhythms of urinary metabolites before and after a week of night shifts. (a) Number of metabolites per class that show a 24-h rhythm only during the day schedule, only during the night schedule, or during both conditions with either altered or similar rhythmicity coefficients. Classification based on differential rhythmicity analysis. (b) Heatmaps visualizing profiles of metabolite classified as rhythmic before (left) and after (right) the series of night shifts. Metabolites are sorted by peak timing. Metabolites identified as rhythmic in both conditions are shown in bold italics. (c) Peak timing of the metabolites classified as rhythmic during the laboratory visit during the day and night schedules. Gray bars represent the scheduled sleep periods during the study visits. (d) Changes in peak timing of the 21 metabolites classified as rhythmic during the day and night schedules. (e) Amplitudes of the metabolites classified as rhythmic during the day schedule and/or night schedule. Gray lines connect metabolites that were rhythmic in both conditions. Data points in c, d, and e represent individual metabolites, and colors represent metabolite classes (colors as in panel a). Abbreviations in panel d: Ac-Orn = acetyl-ornithine; α-AA = α-aminoadipic acid; pHHA = p-hydroxyhippuric acid; But. acid = butyric acid; MH = methylhistidine; Succ. acid = succinic acid; Gly = glycine; Thr = threonine; Tyr = tyrosine.

Summarizing the differential rhythmicity analysis (Figure 3a), we found that 43 metabolites (54%) were classified as rhythmic during the day schedule, compared with 26 (33%) during the night schedule, indicating a significant reduction in the number of rhythmic metabolites (γ2(1) = 6.6, *p* = 0.0103, test of equal proportions). Zooming in on different metabolite classes, this reduction could partially be attributed to a significant decrease in the number of rhythmic organic acids during the night schedule, while no significant change was found for the other metabolite classes (Table 1).

**Table 1.** Difference in number of rhythmic metabolites per metabolite class in the day and night schedule.

Metabolite Class (Total Number)^a^	Rhythmic in Day Schedule (*n*)	Rhythmic in Night Schedule (*n*)	χ^2^ (*df* = 1)	*p* value^b^
Acylcarnitines (*n* = 20)	17	12	2.01	0.157
Amino acids (*n* = 22)	8	7	<0.0001	1
Biogenic amines (*n* = 17)	4	3	<0.0001	1
Organic acids (*n* = 17)	13	4	7.53	**0.006**

Significant *p*-values are shown in bold.

Abbreviations: *df* = degrees of freedom.

a Three metabolites (glucose, choline, and trimethylamine N-oxide) did not belong to any class and were not included in these analyses.

b Test of equal proportions.

Twenty-one metabolites were classified as rhythmic during both conditions, of which 9 displayed similar rhythmicity (i.e., no change in amplitude and peak timing relative to clock time despite the shifted timing of the sleep-wake cycle). These 9 metabolites were exclusively acylcarnitines, which represents a significant overrepresentation of this metabolite class (Table 2). Twelve metabolites displayed altered rhythmicity (i.e., change in amplitude and/or peak timing relative to clock time). No metabolite classes were significantly enriched among these 12 metabolites (Table 2).

**Table 2.** Overrepresentation analysis of metabolite classes.

Category	Acylcarnitines (Total *n* = 20)		Amino Acids (Total *n* = 22)		Biogenic Amines (Total *n* = 17)		Organic Acids (Total *n* = 17)	
	*n*	*p* value	*n*	*p* value	*n*	*p* value	*n*	*p* value
Similar^a^ (*n* = 9)	9	**<0.0001**	0	1.00	0	1.00	0	1.00
Altered^a^ (*n* = 12)	2	0.869	4	0.442	2	0.789	4	0.234
Increased levels (*n* = 13)^b^	7	**0.016**	2	0.932	0	1.00	3	0.567
Decreased levels (*n* = 5)	1	0.778	1	0.814	3	0.639	0	1.00

Significant *p*-values are shown in bold.

a Metabolites classified as rhythmic during both the day and night schedule. “Similar” refers to metabolite profiles with unchanged amplitude and clock time of the peak timing during both conditions; “altered” refers to metabolite profiles with changed amplitude and/or peak timing.

b One metabolite with increased levels (glucose) did not belong to any metabolite class but was counted toward the total number of metabolites in this category.

Further characterization of the cosinor coefficients of the metabolites classified as rhythmic during the day and night schedule (visualized in heatmaps in Figure 3b **)** shows that their peak times are nonuniformly distributed around the 24-h day during both conditions (both *p* < 0.0001, Rayleigh test of uniformity, Figure 3c). As shown in Figure 3c, the metabolite peak times relative to the sleep-wake cycle had changed during the night schedule compared with the day schedule: while during the day schedule the average peak time of rhythmic metabolites occurred at 1535 h (i.e., in the middle of the day-oriented wake period), the average peak time was 0909 h during the night schedule (i.e., at the end of the night-oriented wake period). Among the metabolites classified as rhythmic in both conditions, the changes in peak timing also reflected this altered relative timing with the sleep-wake cycle (Figure 3d). However, no significant difference was observed in the amplitude of the diurnal rhythm among these metabolites between both conditions (mean log_10_ amplitude day schedule: 0.178 vs night schedule: 0.163, *t*(24) = −1.1, *p* = 0.278; mixed-effects model with “metabolite” included as random effect; see Figure 3e).

### Changes in Average 24-h Metabolite Levels

**Figure 4.**
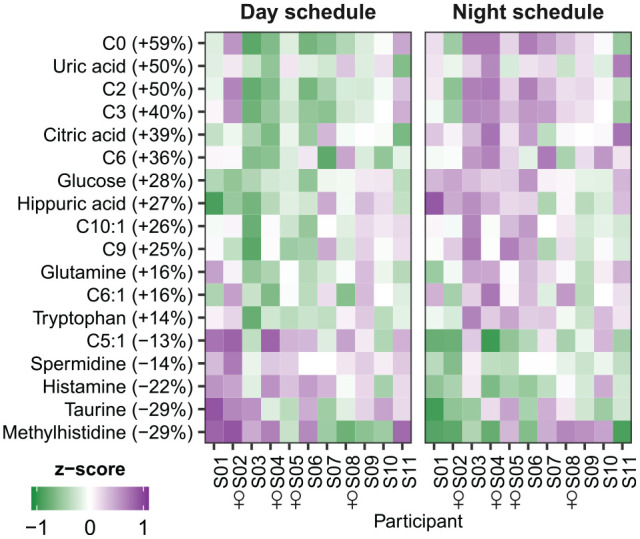
Metabolites classified as showing an increase or decrease in average levels. Heatmap of data shown as *z*-scored values per participant. Metabolites are ordered by the magnitude of change. Percentages behind metabolite names indicate the relative change in metabolite levels from the day schedule to the night schedule.

Furthermore, differential rhythmicity analysis indicated that 18 metabolites displayed a change in mesor (i.e., average levels over 24 h): excretion of 13 metabolites was increased in the night schedule compared with the day schedule, while excretion of 5 metabolites was decreased (Figure 4). Acylcarnitines were significantly overrepresented among the metabolites showing increased levels on the night compared with the day schedule, while no metabolite classes were significantly overrepresented among metabolites with decreased levels (Table 2).

## Discussion

In this study, we provided a detailed characterization of the daily variation in urinary metabolome in a group of police officers before and after a week of working nights. Our main finding is that the rhythmicity of the urinary metabolome had changed when workers lived on a night schedule compared with when they lived on a day schedule, with most notable changes observed for organic acids and acylcarnitines. This is evident from the differential rhythmicity analysis, which showed that significantly fewer metabolites displayed a 24-h rhythm in their urinary levels during night schedule. Furthermore, about half of the metabolites that were classified as rhythmic during both conditions had not regained their relative timing with respect to the shifted sleep-wake cycle during the night schedule compared with the day schedule. Overall, our findings indicate that night shifts induce a state of misalignment of 24-h rhythms in the urinary metabolome in rotating shift workers.

The urinary excretion of a relatively large number of metabolites displayed 24-h rhythms during the day schedule (*n* = 43; 54% of all detected metabolites). This number is comparable to a prior study in which a significant effect of time of day was found in 46% of urinary metabolites (Kim et al., 2014), but it is considerably higher than what was observed by Giskeodegard et al. (2015), who studied 24-h rhythms in a set of urinary metabolites partially overlapping with ours and found that only 5 (16%) out of 32 urinary metabolites showed a 24-h rhythm under entrained conditions (e.g., comparable to our day schedule). However, in that study, principal component analysis revealed large time-of-day variations across the entire metabolomic dataset (Giskeodegard et al., 2015), suggesting that time of day is a considerable source of variation in urinary metabolomic analyses.

Importantly, our study shows that fewer metabolites presented a 24-h rhythm during the night schedule (*n* = 26; 32% of all detected metabolites), an observation that could be due to several factors such as partial weakening of individual rhythms, increased variability of rhythmicity among individual workers, or changes in the timing of masking effects when working nights. This is in contrast to the results of 2 controlled laboratory studies that investigated the effect of simulated night shifts on the plasma metabolome in healthy volunteers, in which similar numbers of metabolites showed a 24-h rhythm in day and night schedules (Skene et al., 2018; Kervezee et al., 2019). Multiple factors potentially give rise to this discrepancy, such as the use of urine instead of blood samples and differences in the response to actual shift work in field settings compared with simulated night shifts in laboratory conditions. Interestingly, organic acids were enriched among the metabolites that were classified as only rhythmic on a day schedule. Organic acids are intermediates of key metabolic processes, including the Krebs cycle, fatty acid oxidation, and protein metabolism (Martinez-Reyes and Chandel, 2020). In addition, they fulfill important cellular signaling roles, such as altering the response of immune pathways and influencing epigenetic mechanisms (Martinez-Reyes and Chandel, 2020). It remains to be investigated whether the loss of 24-h rhythmicity of organic acids during a night schedule also affects the regulation of these downstream processes, although this observation would be consistent with the metabolic and immune disturbances associated with night shifts (Puttonen et al., 2011; Cuesta et al., 2016; Loef et al., 2019; Kervezee et al., 2020; Ruiz et al., 2020).

Acylcarnitines seemed insensitive to shifted behavioral and food intake cycles, as the 9 metabolites that were classified as rhythmic with similar peak times and amplitudes in both conditions consisted exclusively of acylcarnitines. These results are in contrast to the 2 metabolomic studies in plasma conducted in simulated night shift conditions, in which it was found that acylcarnitines were overrepresented among the metabolites that were only rhythmic during the night schedule (Skene et al., 2018; Kervezee et al., 2019). Similarly, we found that acylcarnitines were significantly overrepresented among the metabolites that had increased levels during the night schedule in the current study, which was not observed in our previous simulated night shift experiment on plasma metabolites (Kervezee et al., 2019). However, the increase in the average levels of urinary acylcarnitines on the night schedule extends the findings by Rotter et al. (2018), who found an association between night shift work and elevated levels of acylcarnitines in urine samples collected upon waking. Acylcarnitines are intermediates of mitochondrial fatty acid oxidation, and an increase in their levels is thought to reflect inefficient mitochondrial function and is associated with insulin resistance (Schooneman et al., 2013). Therefore, the finding that acylcarnitine excretion is increased following a week of night shift work lends real-world support for the alterations in fatty acid metabolism and insulin sensitivity that has been observed during circadian misalignment (Wefers et al., 2018). Given the relevance of acylcarnitines to the development of insulin resistance, along with the increased risk of developing type 2 diabetes associated with night shift work, this is an important area for future research.

Like acylcarnitines, glucose was also among the metabolites whose average 24-h levels were higher following night shifts, with an increase of 28%. In our study population, consisting of healthy participants with presumably normal renal function, the increased levels of glucose in urine likely reflect elevated plasma glucose levels (Cowart and Stachura, 1990). Because urinary metabolite levels reflect metabolite excretion over the time period between 2 voids, rather than a single time-point concentration, we are not able to determine whether the increase in urinary glucose levels following night shifts is the result of higher post-prandial glucose excursions or higher fasting levels in plasma. Nevertheless, our observation is consistent with prior controlled laboratory studies as well as field studies, which have shown that night shifts are associated with increased plasma glucose levels and decreased insulin sensitivity (Scheer et al., 2009; Morris et al., 2016b; Proper et al., 2016; Sharma et al., 2017; Wefers et al., 2018; Mason et al., 2020). Furthermore, our study suggests that urine can be used as a non-invasive biofluid to detect acute metabolic responses associated with night shift work.

The finding that, after a week of working nights, different subsets of metabolites are no longer rhythmic, remain aligned to day-oriented behavioral cycles, or (partially) change their peak timing along with the shifted behavioral cycles may reflect changes in the timing of meals or other environmental factors and/or circadian disturbances in the metabolic processes they are involved in. Circadian misalignment includes desynchrony of circadian rhythms in physiology with external timing cues, such as the light/dark cycle or fasting/feeding cycles, as well as internal misalignment of circadian rhythms among cells, tissues, and/or organs (Vetter, 2020). Previous studies that investigated the effect of a simulated night shift protocol on the plasma metabolome showed that under those conditions, 24-h rhythms in plasma metabolites are primarily driven by behavioral cycles (e.g. sleep-wake cycles or meal timing) rather than by the endogenous circadian timing system (Skene et al., 2018; Kervezee et al., 2019). This does not seem to be the case in the current study: the peak timing of several acylcarnitines did not change along with the behavioral cycles, and in general, there were a reduced number of metabolites classified as rhythmic following night shift work. This is in line with prior analyses that were conducted in the same participants as the current study, which revealed that 24-h rhythms in central circadian clock markers (urinary 6-sulfatoxymelatonin and salivary cortisol) had partially adjusted to the shifted behavioral cycles following the week of night shifts, while daily variation in peripheral circadian clock markers (core clock genes) was blunted (Koshy et al., 2019). Therefore, the metabolomic changes that we observed in response to night shift work may be due to impaired regulation of metabolic processes by central and/or peripheral clocks. This raises the question of how the central clock, different peripheral clocks, and environmental factors such as food intake, sleep, or physical activity work in concert to produce these metabolite rhythms under entrained conditions. In addition, the contribution of peripheral clocks in different tissues to the altered rhythmicity observed in different biofluids on a night schedule warrants further investigation.

Besides night shift work by itself, several other factors may underlie the observed changes following the week of night shifts. This includes sleep deprivation associated with night shift work, which has been shown to affect the urinary metabolome (Giskeodegard et al., 2015). Indeed, sleep deprivation was shown to result in increased levels of acylcarnitines in plasma in controlled laboratory studies in healthy human participants (Davies et al., 2014; Weljie et al., 2015; van den Berg et al., 2016). However, the actigraphy-derived sleep and wake times of our study population, as reported in Koshy et al. (2019), suggest that the extent of sleep restriction due to night shift work was relatively small (i.e., on average less than 1 h) compared with previous studies, in which the sleep period was restricted to 4 h or less. Therefore, it is unlikely that sleep deprivation had a major impact on our results, a conclusion corroborated by a recent review that concluded that time of day has a larger impact than sleep disturbances on the urinary metabolome (Hancox et al., 2021).

Furthermore, meal timing may have influenced our results (Kim et al., 2014). In our protocol, meal timing was nearly inverted on the night compared with the day schedule. Although participants did not document their meal timing during the ambulatory periods, we have shown previously, in a different subset of our police officers cohort, that caloric intake occurred later and was dispersed over a longer eating window during night shift days than during rest days or days with other types of shift, while there was no difference in macronutrient composition (Kosmadopoulos et al., 2020). It is probable these findings also apply to the police officers included in the current study and that substantial changes in feeding behavior occurred on their days working nights. Changes in meal timing and other environmental factors may have directly influenced the daily metabolite rhythms, thereby masking endogenous circadian rhythms, as was recently shown in the context of clinical metabolic biomarkers in simulated night shift protocols (Grant et al., 2021). Alternatively, meal timing may also act as a timing cue capable of resetting peripheral clocks, which was previously shown in rodents (Damiola et al., 2000; Stokkan et al., 2001) and possibly also in humans (Krauchi et al., 2002; Wehrens et al., 2017; Lewis et al., 2020). Although we are unable to distinguish between these possibilities with the current study design, it is interesting to note that while the peak timing of various rhythmic metabolites (including mostly organic acids and amino acids) had changed along with the behavioral rhythms on the night schedule, the rhythmic profile of various acylcarnitines had not, giving rise to the possibility that the 24-h rhythms in these metabolites are endogenously generated.

The analyses in this study were performed relative to clock time, thereby providing insight into the temporal dynamics of the metabolome in response to a change in the timing of the rest-activity cycle. However, in line with previously published laboratory studies, it would have been interesting to analyze the metabolite profiles relative to the endogenous circadian phase of each participant. Although we analyzed urinary 6-sulfatoxymelatonin in these participants previously, which can serve as a marker of the central circadian clock, we were unable to obtain reliable phase estimates on an individual level as the individual profiles were significantly rhythmic in only a small subset of participants (Koshy et al., 2019). This limitation reflects the challenging nature of field studies and highlights the need for the development of robust circadian biomarkers in real-world settings, for example, using a single-sample approach or physiological monitoring with wearable technologies.

An important asset of our study is the inclusion of actual shift workers as research participants, providing insight into the metabolomic changes that occur during night shift work in a real-life setting as opposed to controlled laboratory conditions. However, it should be noted that the study population was restricted to police officers on patrol, an occupation characterized by specific physical and psychological demands. Therefore, the question remains as to what extent the results are generalizable to other occupational groups that are involved in night shift work, such as those in health care or industrial settings. Similarly, it is difficult to account for chronic effects of night shifts or for potential incomplete resetting of circadian rhythms since the prior shifts in the current study design. To disentangle these effects, it would be interesting to compare 24-h metabolite rhythms in chronic versus recent shift workers or to shift work-naïve individuals in future studies. More generally, this highlights the need for more controlled laboratory studies that investigate 24-h rhythms in urinary metabolites under baseline and shifted conditions.

Furthermore, in our study, we used urine sampling as a non-invasive and practical approach to collect a comprehensive metabolomic data set. This is especially relevant in the context of using metabolomics to predict circadian phase based on a single biological sample, which, using plasma as a biofluid, recently yielded promising results (Cogswell et al., 2021). However, despite its practicality, an important point of consideration when using urine for these types of analyses is the need for a suitable normalization method to account for differences in urinary flow rate. Creatinine normalization is routinely used but less appropriate since creatinine excretion may display diurnal variation—although this was not observed in the current study—and show substantial interindividual variation (Hancox et al., 2021). Taking advantage of the fact that urine volumes and voiding times were precisely documented during both study visits, we were able to calculate the urinary flow rate of each sample and use this to normalize metabolomic concentrations. This has been deemed the most accurate normalization method, although it requires documenting the volume and voiding times of each sample, which could be impractical in most research settings (Zhou et al., 2006). Therefore, future studies into urinary metabolomics could consider the use of alternative normalization methods (Wu and Li, 2016).

Overall, our results provide insight into the metabolomic response to night shift work. Organic acids and acylcarnitines, intermediates of key metabolic pathways, were most notably affected, possibly reflecting altered mitochondrial function. As with other -omics studies, this work has generated specific hypotheses that can be tested in future studies, for instance, regarding the effect of night shift work on fatty acid oxidation and mitochondrial function. Further research is warranted to explore to what extent the observed metabolomic changes reflect, or contribute to, the adverse metabolic effects associated with night shift work.

## Supplemental Material

sj-pdf-1-jbr-10.1177_07487304221132088 – Supplemental material for The Effect of Night Shifts on 24-h Rhythms in the Urinary Metabolome of Police Officers on a Rotating Work Schedule Supplemental material, sj-pdf-1-jbr-10.1177_07487304221132088 for The Effect of Night Shifts on 24-h Rhythms in the Urinary Metabolome of Police Officers on a Rotating Work Schedule by Laura Kervezee, Anna Koshy, Nicolas Cermakian and Diane B. Boivin in Journal of Biological Rhythms

sj-xlsx-2-jbr-10.1177_07487304221132088 – Supplemental material for The Effect of Night Shifts on 24-h Rhythms in the Urinary Metabolome of Police Officers on a Rotating Work Schedule Supplemental material, sj-xlsx-2-jbr-10.1177_07487304221132088 for The Effect of Night Shifts on 24-h Rhythms in the Urinary Metabolome of Police Officers on a Rotating Work Schedule by Laura Kervezee, Anna Koshy, Nicolas Cermakian and Diane B. Boivin in Journal of Biological Rhythms
