# Supplementary material for: The Effect of Night Shifts on 24-h Rhythms in the Urinary Metabolome of Police Officers on a Rotating Work Schedule
Source: J Biol Rhythms. 2022 Nov 8;38(1):64–76. doi: 10.1177/07487304221132088 (PMC9902972; doi:10.1177/07487304221132088)
Supplement: sj-pdf-1-jbr-10.1177_07487304221132088 – Supplemental material for The Effect of Night Shifts on 24-h Rhythms in the Urinary Metabolome of Police Officers on a Rotating Work Schedule [file sj-pdf-1-jbr-10.1177_07487304221132088.pdf]

Supporting information

Kervezee et al. - The effect of night shifts on 24-h rhythms in the urinary metabolome of police officers on a rotating work schedule

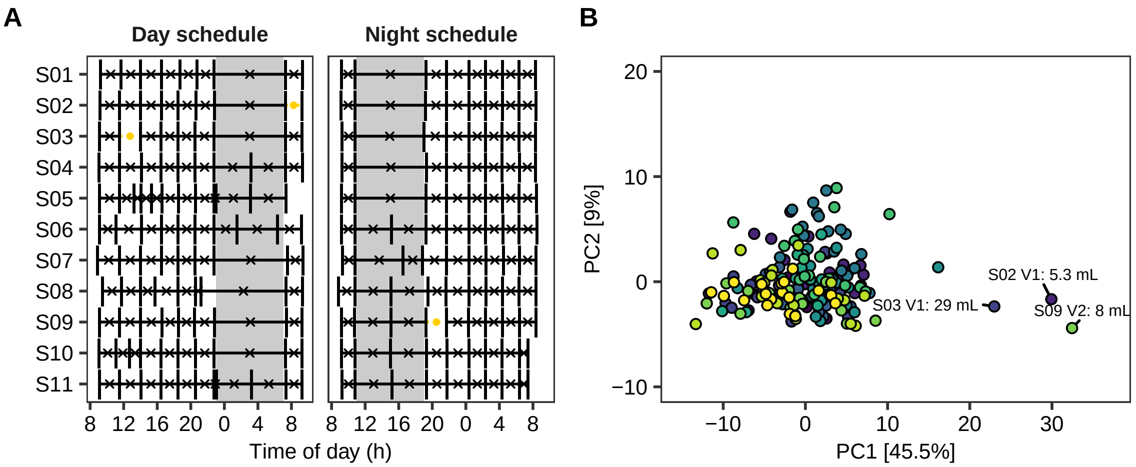

Supplemental Figure S1. A) Overview of the time points of urine collection per participant (S01 – S11). Vertical bars indicate the timing of urine voids per participant during the study visits, cross symbols represent the midpoints between urine collection times. The three samples excluded as outliers from further analysis are shown in orange circles. Grey rectangles represent the timing of the sleep period during the two study visits. **B)** Principal component analysis of the normalized metabolomics data (see Methods for a description of the normalisation procedure). Data points represent individual samples. Axes show the first and second principal components (PC), accounting for 46% and 9% of the variation in the data, respectively. Text labels mark the three samples excluded as outliers from further analyses and indicate their sample volumes as described in the main text. Symbol colors represent different participants.

Supplemental Data S1. Overview of differential rhythmicity results (xlsx file)
